# Heterostructured Nanocrystal Synthesis with Large Lattice Mismatch by Sacrificial Agent Assisted Method

**DOI:** 10.1002/smsc.202500443

**Published:** 2025-10-11

**Authors:** Feng Qin, De‐Ming Liu, Guo‐Yang Chen, Jia‐Xu Yan, Lei Liu, De‐Zhen Shen

**Affiliations:** ^1^ State Key Laboratory of Luminescence Science and Technology Changchun Institute of Optics Fine Mechanics and Physics Chinese Academy of Sciences Changchun 130033 China; ^2^ Center of Materials Science and Optoelectronics Engineering University of Chinese Academy of Sciences Beijing 100049 China; ^3^ National Key Laboratory of Opto‐Electronic Information Acquisition and Protection Technology Anhui University Hefei 230601 China

**Keywords:** heterointerface, heterostructured nanocrystal, NaYF_4_, upcovnersion nanoparticle

## Abstract

Heterostructured nanocrystals (HNCs) integrating dissimilar materials offer unprecedented functionalities for optoelectronics and bioimaging, yet their synthesis remains constrained by severe lattice mismatch (>5%) between crystallographically incompatible phases. To overcome this challenge, a sacrificial agent‐assisted method is introduced to fabricate high‐quality HNCs from materials with incompatible crystal structures and extreme lattice mismatches. Using ZnO nanocrystals as a sacrificial oxygen source, the method demonstrates the epitaxial growth of NaYF_4_/YOF HNCs—combining hexagonal phase NaYF_4_ and cubic phase YOF with a bulk lattice mismatch of 36%. This strategy suppresses shell self‐nucleation and enables facet‐selective heteroepitaxy by maintaining an ultra‐low monomer concentration. Atomic‐resolution characterization reveals a coherent interface between NaYF_4_ {100} and YOF {311} planes, reducing the interfacial mismatch to 7.6%. This method operates efficiently across a wide temperature range (300–320 °C) with >92% yield, while morphology is tunable via Na^+^ additives. This approach facilitates the design of advanced metal fluoride/oxide HNCs for photonics, sensing, and catalysis, offering new opportunities for applications where lattice mismatch has previously limited heterostructure development.

## Graphical abstract

Severe lattice mismatch has prevented the integration of different crystalline phases, resulting in limitations in material selection. This work overcomes the 5% barrier of lattice mismatch in heterostructured nanocrystals. Despite a 36% mismatch, a strategy is developed to construct precise NaYF_4_/YOF heterostructures, achieving a coherent interface and >92% yield. This enables new designs in photonics and catalysis.

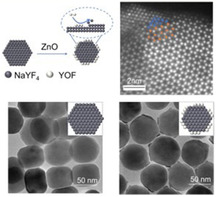

## 1 Introduction

Heterostructured nanocrystals (HNCs) represent a cutting‐edge design strategy that overcomes the inherent limitations of conventional nanocrystals in terms of compositional tunability and geometric features. This is achieved by coordinating the control of the size, morphology, and crystal phase of constituent units, coupled with the engineering of their spatial arrangement and interface structure, collectively enabling enhanced performance and multifunctionality.^[^ ^1^, ^2^, ^3^, ^4^, ^5^, ^6^ ^]^ Consequently, HNCs demonstrate exceptional promise in frontier domains including clean energy,^[^ ^7^, ^8^ ^]^ bioimaging,^[^ ^9^, ^10^ ^]^ and optoelectronic devices.^[^ ^11^ ^]^ In particular, some high‐quality HNCs, such as β‐NaYF_4_/NaGdF_4_,^[^ ^12^, ^13^ ^]^ α‐NaYF_4_/CaF_2,_^[^ ^14^, ^15^ ^]^ and CdSe/CdS,^[^ ^16^, ^17^ ^]^ have been successfully synthesized and have exhibited enhanced luminescence or unique photoelectric properties. These high‐quality HNCs are mainly synthesized by the seed‐mediated growth method,^[^ ^18^, ^19^ ^]^ which generally requires the seed and shell materials to have a similar crystalline structure, with moderate lattice mismatches (<5%).^[^ ^20^, ^21^ ^]^ However, most nanocrystals have different crystalline structures with quite larger lattice mismatches. The large lattice mismatches will reduce the controllability of HNCs growth, which causes the self‐nucleation issue of the shell materials. More importantly, the performance of HNCs is highly dependent on precise controllability during growth. This involves achieving selective growth of heterostructures on specific crystal facets and precisely regulating the growth extent, thereby ensuring the reliability of the material performance. Therefore, solving this lattice mismatch constraint problem is essential for synthesizing more high‐quality HNCs with large lattice mismatches.

Unlike the seed‐mediated growth method, cation exchange^[^ ^22^, ^23^, ^24^ ^]^ offers an indirect pathway for synthesizing HNCs. This approach uses ion substitution mechanisms to directly reconfigure the crystal lattice framework, enabling the construction of more complex HNCs.^[^ ^25^, ^26^ ^]^ Worth to note that Zhang et al. reported an innovative ion exchange method by applying an amorphous Ag_2_S shell as a transition layer, which successfully synthesized noble metal and II–VI semiconductors HNCs with large lattice mismatch (43%) (Au/CdS).^[^ ^27^ ^]^ However, this method is not suitable for metal fluorides and metal oxides, due to the higher electronegativity of these materials hindering the formation of the sulfide transition shell. Therefore, the lattice mismatch constraint urgently needs to be addressed for synthesizing high quality HNCs with large lattice mismatches, especially for metal fluorides and metal oxides.

**Scheme 1:**
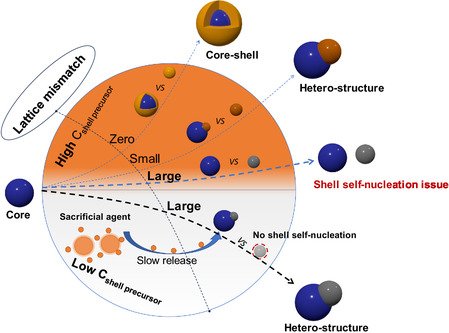
Heterostructure growth with different Lattice mismatch. During heterostructure growth under conditions of significant lattice mismatch, the presence of high concentrations of shell precursors can induce self‐nucleation of the shell material, resulting in growth failure. To address this issue, a low‐concentration shell growth strategy is proposed. This approach utilizes sacrificial agents to replace the conventional practice of direct precursor addition. The utilization of sacrificial agents enables the achievement of slow release of shell‐forming materials and the promotion of successful formation of heterostructures.

Here, we present a new synthesis strategy for NaYF_4_/YOF HNCs to overcome the limitation of the lattice mismatch constraint. Different from the traditional seed‐mediated growth method of directly inputting the shell material, we applied ZnO nanocrystals as a sacrificial agent to gradually supply the shell materials (see **Scheme** **1**), which not only efficiently avoided the self‐nucleation issue of shell material, but also promoted the facet‐selective heterogeneous epitaxial growth of NaYF_4_/YOF HNCs by providing an extremely low monomer concentration condition. Furthermore, a high production rate was achieved without additional purification steps, because the extra sacrificial agent can be easily removed by adding a small amount of oleic acid after the reaction.

## 2 Results and Discussion

**Figure 1:**
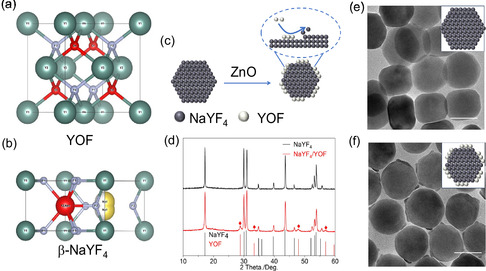
a) Crystal unit cell of YOF (cubic phase, space group: Fm$\bar{3}$m (225), lattice parameter *a* = 5.37 Å). b) Crystal unit cell of β‐NaYF_4_ (hexagonal phase, space group: P6_3_ m^−1^ (176), lattice parameters *a* = 5.96 Å, *c* = 3.53 Å). c) Schematic illustration of the synthesis of NaYF_4_/YOF HNCs driven by a ZnO sacrificial agent acting as an oxygen source. d) XRD patterns of β‐NaYF_4_ seeds (JCPDS: 16‐0334) and NaYF_4_/YOF HNCs (YOF phase, JCPDS: 06‐0346). TEM images of e) NaYF_4_ and f) NaYF_4_/YOF HNCs. Scale bars: 50 nm.

Structural analysis reveals a significant lattice mismatch between the two distinct crystal systems, with a minimum value of 36% observed among their low‐index crystal planes ({100}, {111}, {001}). This value, detailed in Table S1, Supporting Information, was specifically calculated for the interface between the (001) plane of hexagonal NaYF_4_ and the (111) plane of cubic YOF. Under these conditions, seed‐mediated growth method typically results in self‐nucleation of YOF (Figure S1, Supporting Information). However, by applying a ZnO sacrificial agent to provide an oxygen source, we successfully synthesized NaYF_4_/YOF HNCs. As shown in **Figure** **1**f, transmission electron microscope (TEM) image demonstrates facet‐selective growth of YOF exclusively on the {100} facets of NaYF_4_ seeds. Moreover, the high‐resolution transmission electron microscope (HR‐TEM) results of HNCs (Figure S2, Supporting Information) synthesized in different batches demonstrate remarkable consistency, collectively confirming the selective epitaxial growth of YOF on the {100} facets of NaYF_4_. X‐ray diffraction (XRD) analysis was used to determine the crystal structure of HNCs, and the results are shown in Figure 1d. The XRD patterns were consistent with the reference patterns for cubic YOF and β‐NaYF_4_ nanocrystals. Notably, the diffraction peaks originating from YOF are relatively weak, which is consistent with its smaller volume fraction within the HNCs.

**Figure 2:**
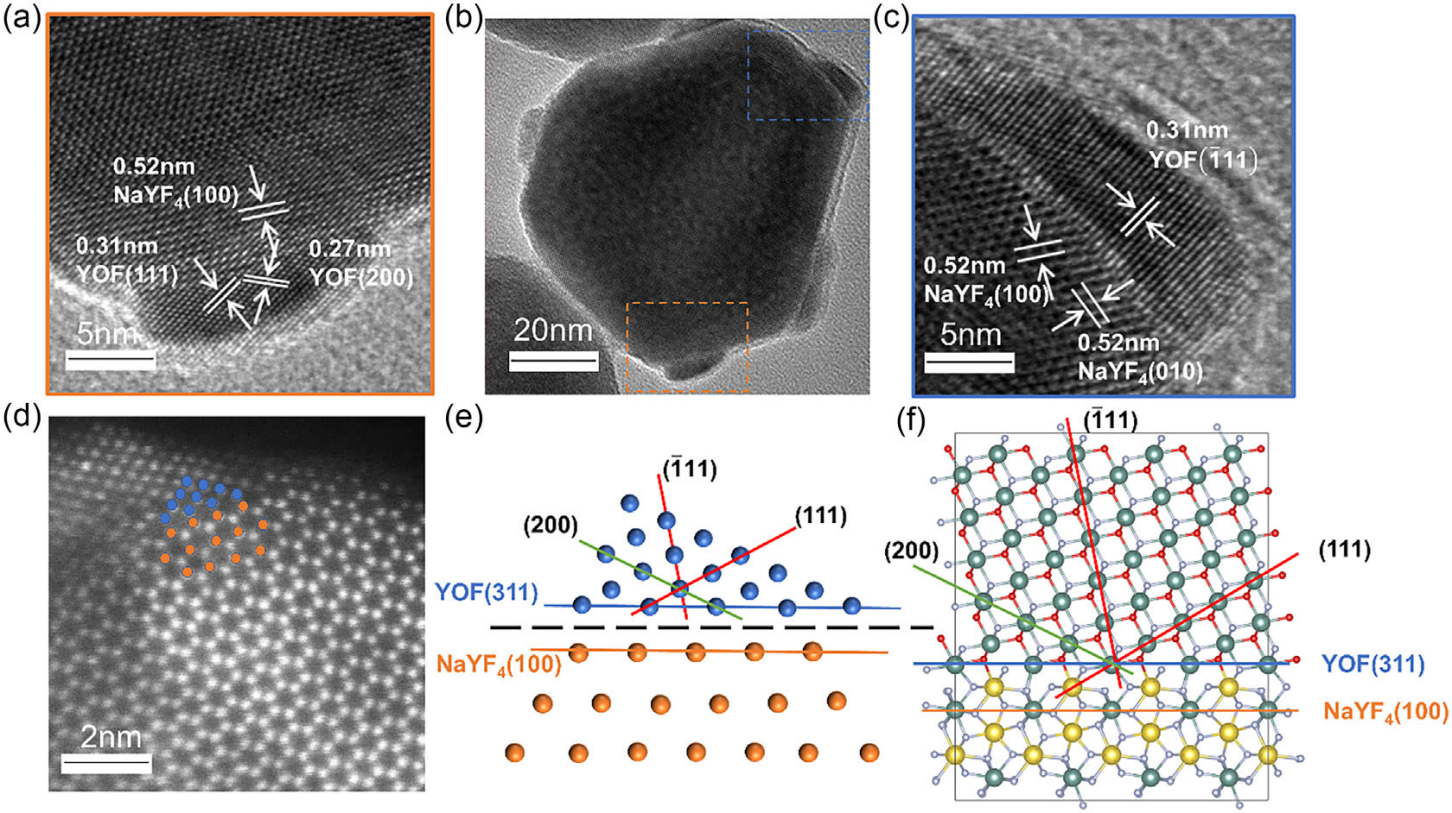
Structural characterization of a single NaYF_4_/YOF HNC. a–c) High‐resolution TEM images of a single nanocrystal: (a,c) show enlarged views of the different box markers in (b). d–e) Atomic‐resolution HAADF‐STEM image of the NaYF_4_/YOF HNC observed along the [001] zone axis of NaYF_4_, which further confirms the interface structure. f) Atomic structure model of the NaYF_4_/YOF HNC with a heterointerface constructed between β‐NaYF_4_ (100) crystal face and YOF (311) crystal face.

To further understand the formation mechanism of the NaYF_4_/YOF HNCs. HR‐TEM analysis was conducted (**Figure** **2**a–c). Figure 2b shows a representative hexagonal NaYF_4_ seed with YOF nanoparticles growing on its six equivalent {100} facets. Figure 2a,c present magnified views of the boxed regions in Figure 2b, revealing detailed crystal structures at the heterointerfaces. In Figure 2a, the measured interlayer spacings of 0.31 nm and 0.27 nm correspond to the (111) and (200) planes of YOF, respectively. Figure 2c further displays the crystal structure of a heterointerface located on a different {100} facet of the same NaYF_4_/YOF HNC. The interlayer spacing of 0.52 nm in the inner region agrees well with the {100} lattice plane of NaYF_4_, while the outer region exhibits an interlayer spacing of 0.31 nm, matching the ($\bar{1}$11) plane of YOF. As is well known, the typical NaYF_4_ nanocrystal is a hexagonal prism surrounded by low‐index lattice planes belonging to the {100} and {001} families. Therefore, it is reasonable to conclude that YOF nanocrystals grown on equivalent {100} facets, such as (100) and (010), have the same crystal structure. Further theoretical analysis of the interplanar angular relationships (Figure S3 and S4, Supporting Information) indicated that the YOF heteroepitaxial plane satisfying the specific angular constraints should be the (311) plane. This inference was experimentally verified by HR‐TEM measurements (Figure 2a,c): The measured angle between the YOF($\bar{1}$11) plane and the heterointerface is ≈80°, while that between the YOF(111) plane and the interface is about 30°, consistent with the theoretical predictions for the (311) plane. Moreover, the lattice orientation of the NaYF_4_(100) plane was found to be parallel to the heterostructure interface. Based on these findings, we conclude that this heterointerface is composed of the NaYF_4_(100) plane and the YOF(311) plane.

To resolve the heterointerface structure at the atomic scale, high‐angle annular dark‐field scanning transmission electron microscope (HAADF‐STEM) imaging was performed along the [001] zone axis of NaYF_4_ (Figure 2d). The positions of the Y atoms at the interface were clearly resolved. Through facet indexing at the heterointerface, it was determined that the (100) plane of NaYF_4_ and the (311) plane of YOF are parallel at the interface (Figure 2e), consistent with the model proposed based on our earlier HR‐TEM analysis. Figure 2f presents the constructed atomic model of the NaYF_4_/YOF HNC, showing the heterointerface formed between the NaYF_4_ (100) and YOF (311) planes. To evaluate the lattice mismatch at the heterointerface, we reconstructed the 2D unit cells of the YOF (311) and NaYF_4_ (100) crystals faces (Figure S7, Supporting Information). The calculated lattice mismatch at the interface is 7.6%, despite the significant lattice mismatch of 36% between hexagonal‐phase NaYF_4_ and cubic‐phase YOF. This result confirms that facet‐selective growth of the NaYF_4_/YOF HNCs was achieved, as the formation of the high‐index YOF (311) plane effectively minimizes the interfacial lattice mismatch.

**Figure 3:**
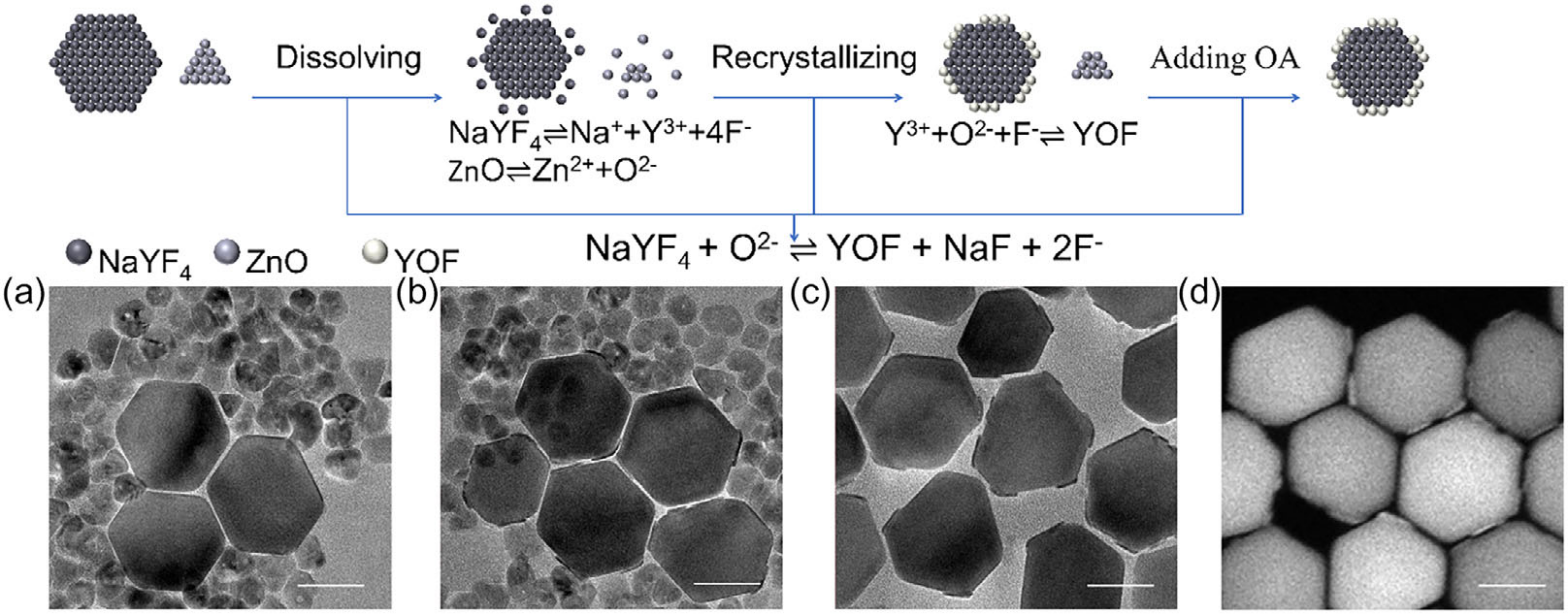
Schematic illustration of the synthetic mechanism for NaYF_4_/YOF HNCs. a–c) TEM images monitoring the reaction progression: (a) Initial mixture of ZnO and NaYF_4_ nanocrystals; (b) intermediate stage showing coexisting ZnO and NaYF_4_/YOF HNCs; and (c) final product of NaYF_4_/YOF HNCs after ZnO removal by oleic acid (OA) treatment. d) STEM image of the product in (c). Scale bars: 50 nm.

To elucidate the synthesis mechanism of NaYF_4_/YOF HNCs, we monitored the morphology changes of NaYF_4_ seeds and ZnO sacrificial agent at different reaction stages. The synthesis scheme and the resulting materials at different stages are illustrated in **Figure** **3**. The results demonstrate that the formation of this heterostructure involves partial dissolution and structural reorganization of NaYF_4_. At the beginning of the reaction (Figure 3a), TEM image showed the presence of prmade ZnO nanocrystals (size: 29 nm, Figure S8, Supporting Information) and NaYF_4_ nanocrystals (size: 93 nm) in the OA‐OAm‐ODE mixed solvent system. As the reaction proceeded (Figure 3b), TEM image revealed the presence of ZnO nanocrystals alongside the newly generated NaYF_4_/YOF HNCs. We propose that the formation of the NaYF_4_/YOF heterostructure originates from O^2−^ ions generated by the dissolution of ZnO, which triggers a recombination reaction on the surface of the NaYF_4_ nanocrystals.^[^ ^28^ ^]^ The proposed reaction mechanism involves the dissolution processes of NaYF_4_ and ZnO nanocrystals: NaYF_4_ ⇌ Na^+^ + Y^3+^ + 4F^‐^ and ZnO ⇌ Zn^2+^ + O^2−^. When the concentrations of O^2−^, Y^3+^, and F^−^ exceed the saturation concentration for YOF formation, the reaction Y^3+^ + O^2−^ + F^‐^ ⇌ YOF occurs, leading to the heteroepitaxial growth of YOF nanocrystals on the NaYF_4_ surface. The overall composite reaction can be summarized as NaYF_4_ + O^2−^ ⇌ YOF + NaF + 2F^‐^. This confirms that ZnO primarily serves as an O^2−^ ion source without directly participating in heterostructure formation, while both fluorine and yttrium sources are derived from the NaYF_4_ nanocrystals themselves. The following experimental results further validate our hypothesis. First, the NaYF_4_ nanocrystals maintained their morphological features throughout this process, with only a slight decrease in size (Figure S9, Supporting Information). Second, Figure S10, Supporting Information shows NaYF_4_ nanocrystals of different sizes: 25, 55, and 120 nm. After growing YOF for 90 min, the average size decreased slightly. This suggests partial dissolution of the NaYF_4_ nanocrystals, releasing F^−^ and Y^3^^+^ ions necessary for NaYF_4_/YOF formation.^[^ ^29^, ^30^, ^31^ ^]^

When the system was naturally cooled to 200 °C, a small amount of Oleic acid (OA) was added to dissolve the residual ZnO nanocrystals.^[^ ^32^, ^33^ ^]^ TEM results (Figure 3c) confirmed the final acquisition of NaYF_4_/YOF HNCs. Control experiments (Figure S11, Supporting Information) showed that the target heterostructure could not be synthesized without the ZnO sacrificial agent. This confirms ZnO's essential role as the source of O^2−^ ions. However, to achieve more precise control over the formation of heterogeneous structures, we must systematically study how reaction conditions, such as temperature, surfactant, and ion concentration, affect the growth process involving sacrificial agents.

**Figure 4:**
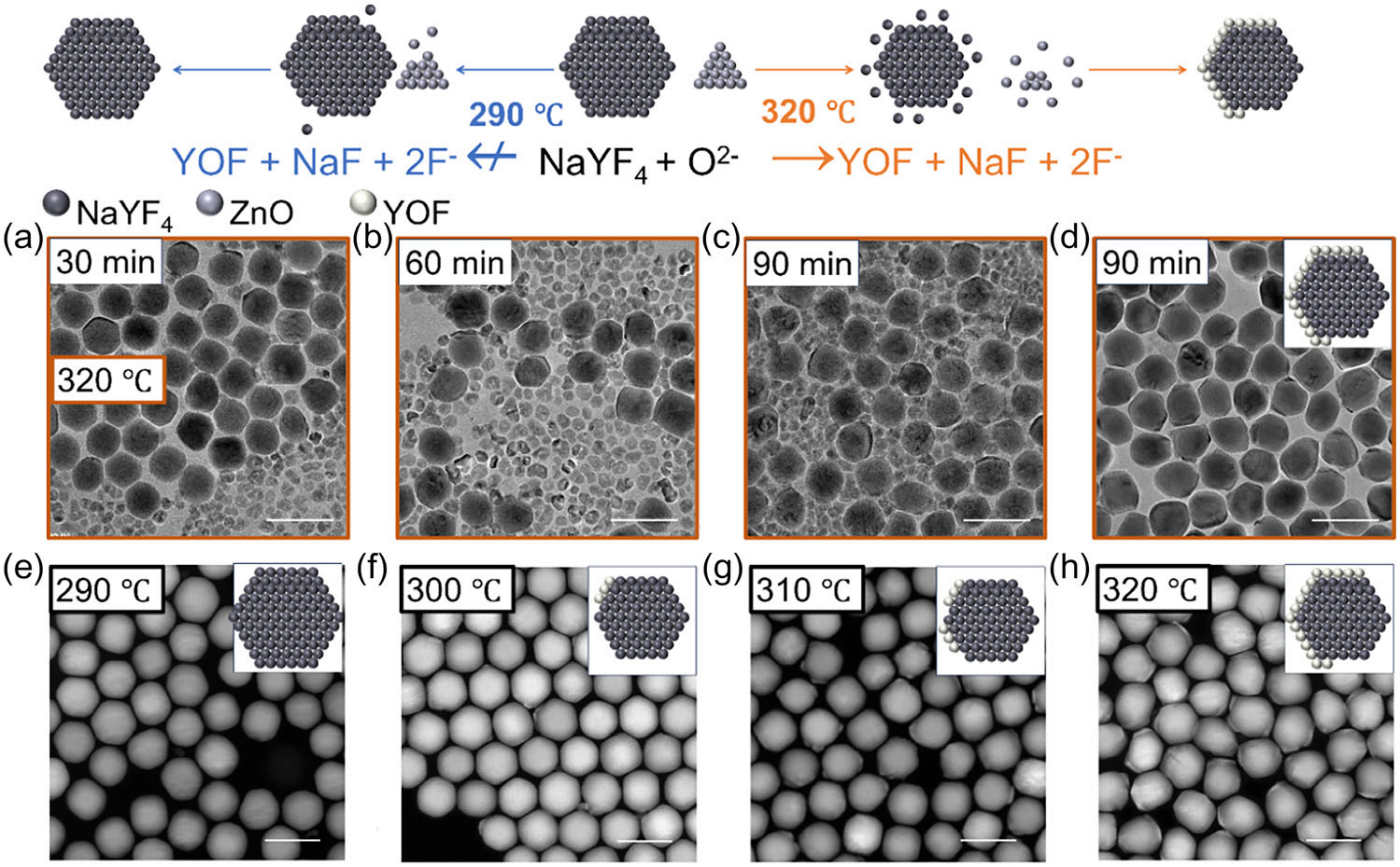
Schematic illustration of NaYF_4_/YOF synthesis at different reaction temperatures. a–c) TEM images of samples collected at different reaction times (30, 60, and 90 min) for the reaction performed at 320 °C. d) TEM image of the product shown in (c) after ZnO removal. e–h) STEM images of the products obtained at different reaction temperatures (290, 300, 310, and 320 °C). Scale bars: 100 nm.

To reveal the growth controllability of NaYF_4_/YOF HNCs by the reaction temperature, we studied the effect of varied temperatures on the productivity of NaYF_4_/YOF HNCs, as shown in **Figure** **4**. We defined the productivity of NaYF_4_/YOF HNCs as the ratio of NaYF_4_/YOF HNCs to the total nanocrystals (estimated by counting 200 nanocrystals). We found the productivity increased markedly with temperature: from 0% at 290 °C (Figure 4e) to 24% at 300 °C (Figure 4f), 59% at 310 °C (Figure 4g), and ultimately reaching 92% at 320 °C (Figure 4h). TEM observations at different reaction times (Figure 4a–d and S12, Supporting Information) confirmed that higher temperatures accelerate heterostructure formation. Additionally, XRD patterns (Figure S13, Supporting Information) revealed that the diffraction peaks of YOF became significantly more intense at higher temperatures, consistent with the enhanced heterostructure productivity confirmed by STEM statistics. This phenomenon can be explained by the fact that an elevated reaction temperature promotes the dissolution of NaYF_4_ seeds and the ZnO sacrificial agent. Although the process accelerates the release of ions, such as Y^3+^, F^−^, and O^2−^, the resulting ion concentrations remain insufficient to induce self‐nucleation of the shell material. Consequently, this process further promotes the heterogeneous nucleation of YOF nanocrystals.

**Figure 5:**
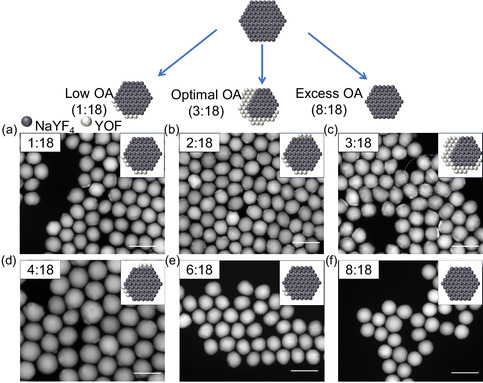
Schematic illustration of the NaYF4/YOF synthesis with varying OA content in OAm‐ODE mixed solvents. a–f) STEM images of NaYF4/YOF heterostructures synthesized at different OA volumes (total volume maintained at 18 mL). Scale bars: 100 nm.

We further studied the effect of OA concentration on the growth of NaYF_4_/YOF HNCs. In this experiment, NaYF_4_/YOF HNCs were cultivated using six groups of oleic acid with varying concentrations. (OA: solvent = 1:18, 2:18, 3:18, 4:18, 6:18, and 8:18, corresponding to **Figure** **5**a–f). The heterostructure productivity initially improves as the OA:solvent ratio increases from 1:18 to 3:18. However, further increase to higher ratios (4:18, 6:18, and 8:18) results in a progressive decline in productivity, ultimately reaching zero. XRD analysis (Figure S14, Supporting Information) confirms this trend, showing first intensified and subsequently weakened YOF diffraction peaks with increasing OA concentration. This is because that OA has two opposite roles in the process of YOF formation. First, it reacts with F^−^ in the solution to make HF, which shifts the equilibrium of the reaction (NaYF_4_ + O^2^^−^ ⇌ YOF + NaF + 2F^−^) to the right and increases the YOF productivity. But OA will also raise the concentration of Zn^2+^ in the solution,^[^ ^34^ ^]^ which will lower the concentration of O^2−^ in the dissolution equilibrium of ZnO (ZnO ⇌ Zn^2+^ + O^2−^). This shifts the reaction (NaYF_4_ + O^2−^ ⇌ YOF + NaF + 2F^‐^) moving to the left and reducing the YOF productivity. So, we saw that productivity was dependent on OA concentration in a nonmonotonic way and the best ratio of OA is 3:18. When there's a medium amount of OA, it helps form heterostructures by capturing free F^−^ ions through HF generation. But when there's too much OA (4:18–8:18), it lowers the O^2−^ ion concentration, which decreases the YOF productivity. Therefore, applying an appropriate amount of OA is a prerequisite for the successful synthesis of NaYF_4_/YOF HNCs.

**Figure 6:**
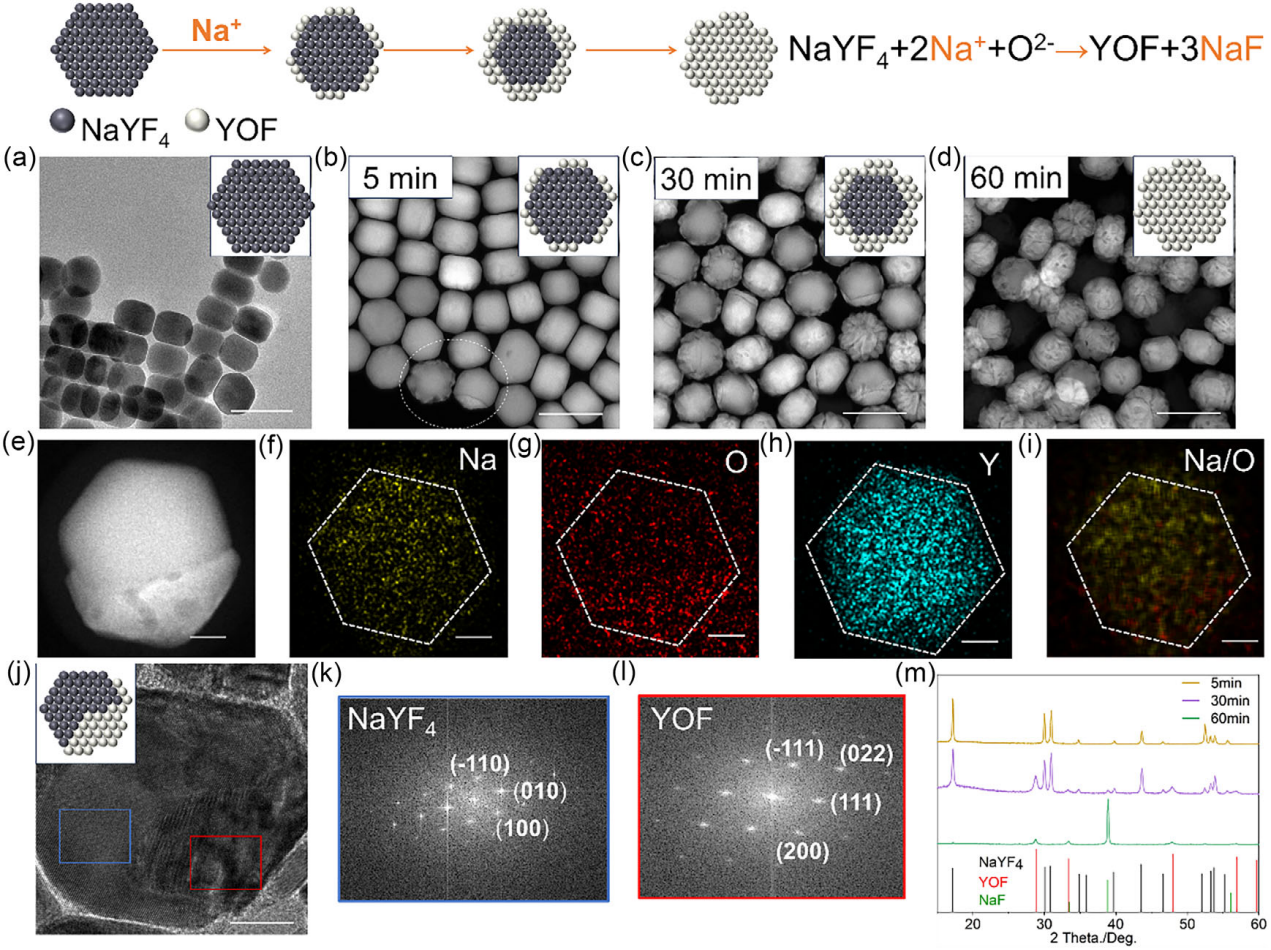
Schematic illustration of morphological control in NaYF_4_/YOF HNCs regulated by Na^+^ additives. a–d) TEM and STEM images collected during the reaction at different time points: (a) NaYF_4_ seeds (TEM), (b) 5 min (STEM), (c) 30 min (STEM), and (d) 60 min (STEM). e) STEM image of the NaYF_4_/YOF HNC with corresponding elemental mapping: f) Na, g) O, h) Y, and i) Na and O. j) HR‐TEM image of the NaYF_4_/YOF HNC. k,l) Fast Fourier transform (FFT) patterns obtained from different regions marked in (j). m) XRD patterns collected at 5, 30, and 60 min during the reaction. Scale bars: 100 nm (a–d) and 20 nm (e–j).

Although temperature and OA concentration significantly influence YOF formation, they offer only limited control over this process. According to the reaction equation (NaYF_4_ + O^2−^ ⇌ YOF + NaF + 2F^−^), lowering the F^−^ ion concentration effectively promotes YOF generation (the reaction can be rewritten as: NaYF_4_ + 2Na^+^ + O^2−^ ⇌ YOF + 3NaF). Therefore, we propose a strategy utilizing excess sodium ions to lower the F^−^ concentration, with the aim of promoting YOF formation. Specifically, during the initial stage (5 min), the primary YOF heterolayer begins to form at the edges of the NaYF_4_ seeds, as observed by STEM (**Figure** **6**b). Upon entering the intermediate stage (30 min), YOF extends further into the interior of the particles. This is evidenced by the progressively intensifying YOF XRD diffraction peaks (Figure 6m) and the STEM mapping results (Figure 6e–i). Concurrently, fast Fourier transform (FFT) patterns obtained from different regions of a single NaYF_4_/YOF heterostructure particle distinctly differentiate the hexagonal phase NaYF_4_ and the cubic phase YOF (Figure 6j–l). Ultimately, after 60 min of reaction, the YOF domains completely replace the NaYF_4_ seeds. This is confirmed by single‐particle HR‐TEM (Figure S16, Supporting Information) and XRD analysis, which detects only YOF and the easily removable NaF byproduct. Therefore, the addition of Na^+^ enables enhanced control over the growth of NaYF_4_/YOF heterostructures.

## 3 Conclusions

In summary, we report a method for synthesizing NaYF_4_/YOF HNCs with large lattice mismatch. This method can be summarized as “$A_{\left({\text{NaYF}}_{4}\right)}{\text{+B}}_{\text{(ZnO)}}\to {\text{A/C}}_{{\text{NaYF}}_{4}\text{/YOF}}$”, where *B*_(ZnO)_ as a sacrificial agent. The chemical equation is: NaYF_4_ + O^2−^ ⇌ YOF + NaF + 2F^‐^. We name this HNC synthesis method “sacrificial agent assisted method”. The use of a sacrificial agent prevents self‐nucleation of the shell material, a common issue encountered in conventional synthesis methods. Additionally, the growth process is highly controllable, successful synthesis can be achieved over a relative wide range of reaction temperature, from 300 to 320 °C. Due to absence of direct shell precursor application, the productivity of NaYF_4_/YOF HNCs can be improved by moderately increasing the reaction temperature and the amount of OA. Meanwhile, the addition of Na^+^ enables enhanced control over the growth of NaYF_4_/YOF heterostructures. This reliable strategy for synthesizing heterostructured nanomaterials can be extended to other rare‐earth fluorides, enriching the existing library of heterocomposite nanomaterials.

## 4 Experimental Section

4.1

4.1.1

##### Materials and Reagent

Yttrium chloride hexahydrate (YCl_3_·6H_2_O, 99.99%), sodium hydroxide (NaOH, 98%), ammonium fluoride (NH_4_F, 99.99%), and zinc acetate dihydrate (Zn(CH_3_COO)_2_·2H_2_O, 99.7%) were purchased from Shanghai Aladdin Reagent Co., Ltd. Oleic acid (OA, 90%), oleylamine (OAm, 80–90%), and 1‐octadecene (ODE, 90%) were purchased from Sigma‐Aldrich. All reagents were used as received without further purification.

##### Synthesis of β‐NaYF_*4*_ *Nanocrystals as Seeds*

A modified synthesis method was employed to prepare β‐NaYF_4_ seeds.^[^ ^35^ ^]^ In a typical procedure, YCl_3_ (1.0 mmol) dissolved in 2 mL of methanol solution was mixed with OA (6 mL) and ODE (15 mL) in a 100 mL three‐neck round‐bottom flask. The mixture was degassed under Ar flow while heating to 160 °C, followed by an isothermal reaction for 30 min to form a clear solution, which was then cooled to room temperature. Subsequently, 10 mL of a methanol solution containing NH_4_F (4 mmol) and NaOH (2.5 mmol) was added to the flask, and the mixture was stirred continuously for 60 min. The solution was slowly heated to 110 °C and maintained isothermally for 30 min to evaporate the methanol and residual water. The reaction mixture was rapidly heated to 300 °C and held for 90 min under isothermal conditions before cooling naturally to room temperature. The resulting nanocrystals were precipitated by adding ethanol, followed by four washing cycles with a mixture of ethanol, methanol, and cyclohexane (volume ratio 1:1:2). Finally, the purified NaYF_4_ nanocrystals were redispersed in 10 mL of cyclohexane for further use.

##### Synthesis of ZnO Nanocrystals as Sacrificial Agents

A modified synthesis method was employed to prepare ZnO nanocrystals.^[^ ^36^ ^]^ In a typical procedure, zinc acetate (1 mmol) was mixed with OA (1 mL) and ODE (10 mL) in a 100 mL three‐neck round‐bottom flask. The mixture was degassed under Ar flow while heating to 160 °C, followed by isothermal reaction for 30 min to form zinc oleate. Subsequently, 6 mL of OAm was injected, and the mixture was stirred continuously for 30 min. The reaction mixture was then rapidly increased to 300 °C and maintained for 60 min under isothermal conditions before cooling naturally to room temperature. The final products were washed and dispersed in 10 mL of cyclohexane as sacrificial agents.

##### Synthesis of NaYF_*4*_ */YOF Heterostructure Nanocrystals*

In a typical procedure, 0.1 mmol of NaYF_4_ nanocrystals, 0.2 mmol of ZnO nanocrystals, 1 mL of OA, 8 mL of OAm, and 9 mL of ODE were loaded into a 50 mL three‐neck round‐bottom flask. The mixture was gradually heated to 120 °C and maintained at this temperature for 30 min to remove residual water and cyclohexane. Subsequently, the reaction temperature was rapidly increased to 315 °C and maintained for 90 min under isothermal conditions. After natural cooling to 200 °C, 3 mL of OA was injected into the flask to dissolve excess ZnO nanocrystals, followed by further cooling to room temperature. The resulting nanocrystals were precipitated by adding ethanol and washed four times with a mixture of ethanol, methanol, and cyclohexane (volume ratio 1:1:2). Finally, the obtained nanocrystals were redispersed in 5 mL of cyclohexane.

##### Characterizations

Standard TEM, HR‐TEM, and HAADF‐STEM images were acquired using a Talos F200s microscope. Elemental mapping images were acquired with the same TEM system equipped with a silicon drift detector (SSD) energy‐dispersive X‐ray spectroscopy (EDS). The samples were prepared for TEM analysis by placing a drop of a dilute suspension of nanocrystals onto carbon‐coated 200‐mesh copper grids, with the detector set at 77 K. Powder XRD patterns were collected using a PANalytical X’Pert Pro MPD diffractometer using Cu Kα1 radiation (40 kV, 40 mA, *λ* = 0.15418 nm). The samples were prepared through multiple cycles of drop‐casting nanocrystal dispersions in cyclohexane onto a wafer and subsequent drying.

## Supporting Information

Supporting Information is available from the Wiley Online Library or from the author.

## Conflict of Interest

The authors declare no conflict of interest.

## Supporting information

Supplementary Material

## Data Availability

The data that support the findings of this study are available from the corresponding author upon reasonable request.
