# Supplementary material for: Heterostructured Nanocrystal Synthesis with Large Lattice Mismatch by Sacrificial Agent Assisted Method
Source: Small Sci. 2025 Oct 11;5(12):e202500443. doi: 10.1002/smsc.202500443 (PMC12697818; doi:10.1002/smsc.202500443)
Supplement: Supplementary file 1 — Supplementary Material [file SMSC-5-e202500443-s001.pdf]

Supporting information

Heterostructured Nanocrystal Synthesis with Large Lattice Mismatch by Sacrificial Agent Assisted Method

Feng Qin[1,2,], De-Ming Liu*[1,2,3], Guo-Yang Chen[1,2], Jia-XuYan[1,2,], Lei Liu[1,2,3] and De-Zhen Shen[1,2,3]

1. State Key Laboratory of Luminescence Science and Technology, Changchun Institute of Optics Fine Mechanics and Physics Chinese Academy of Sciences, Changchun,130033 , China

2. Center of Materials Science and Optoelectronics Engineering, University of Chinese Academy of Sciences, Beijing 100049, China.

3. National Key Laboratory of Opto-Electronic Information Acquisition and Protection Technology, Anhui University, Hefei, 230601, China.

[*] *Email: liudeming0715@gmail.com*

Table 1. The mismatches are calculated by the conventional definition given by $\frac{\text{Shell lattice parameter-Core lattice parameter}}{\text{Core lattcie parameter}}$.

β-$NaYF_4$	(100)	(001)
YOF	(100)	(111)
Mismatch(%)	52%	-36%

Given that the primary exposed facets of hexagonal-phase $NaYF_4$ are the prismatic {100} planes and basal (001) plane, we calculated the lattice mismatch with cubic-phase YOF by comparing interatomic spacings across two facet pairs:

1. $NaYF_4$ {100} vs. YOF {100}:

Compared parameter: Interatomic spacing along the c-axis of $NaYF_4$ (3.53 Å) vs. that along the a-axis of YOF (5.37 Å).

Mismatch: (5.37-3.53)/3.53≈ 52%

2. $NaYF_4$ (001) vs. YOF {111}

Compared parameter: In-plane interatomic spacing on the $NaYF_4$ basal plane (a = 5.96 Å) vs. nearest-neighbor atomic spacing on the YOF {111} plane ($\sqrt{2}a/2 \approx 3.79$ Å)

Mismatch: (3.79-5.96)/5.96≈ -36%

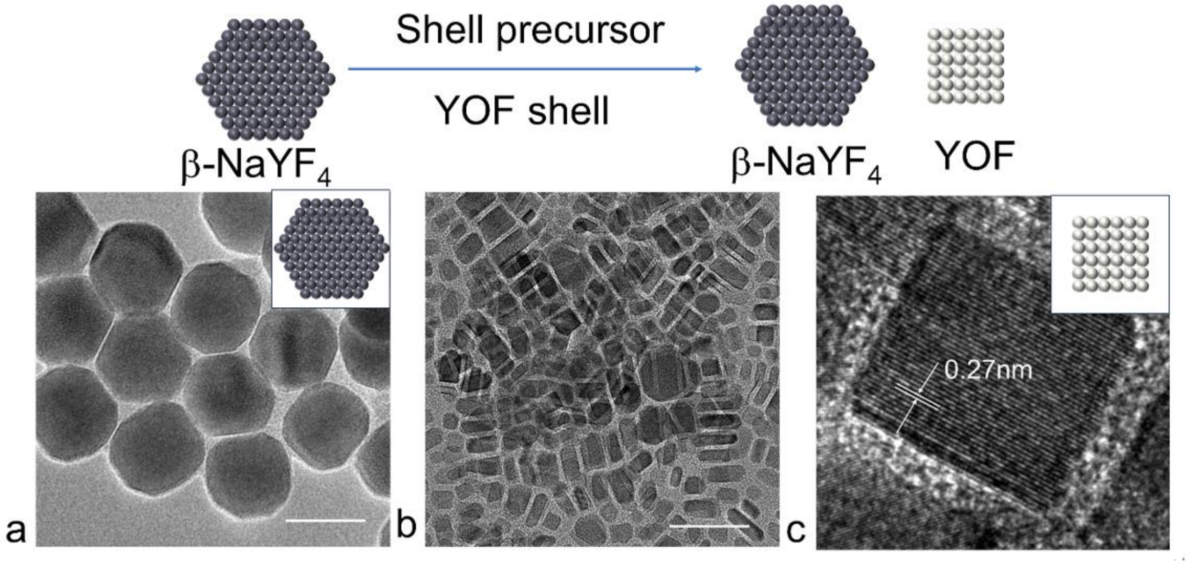

Figure S1. Seed-assisted growth method was used to grow $NaYF_4$/YOF HNCs. (a) TEM images of $NaYF_4$ seeds. (b) $NaYF_4$/YOF HNCs were grown by directly adding yttrium oleate and ammonia fluoride to an OAm-OA-ODE solution and then reacting the mixture at 300 °C for 90 minutes. The TEM image shows that many YOF nanocrystals underwent self-nucleation. (c) HR-TEM image of YOF shows that the exposed crystal plane of YOF is the (100) plane.

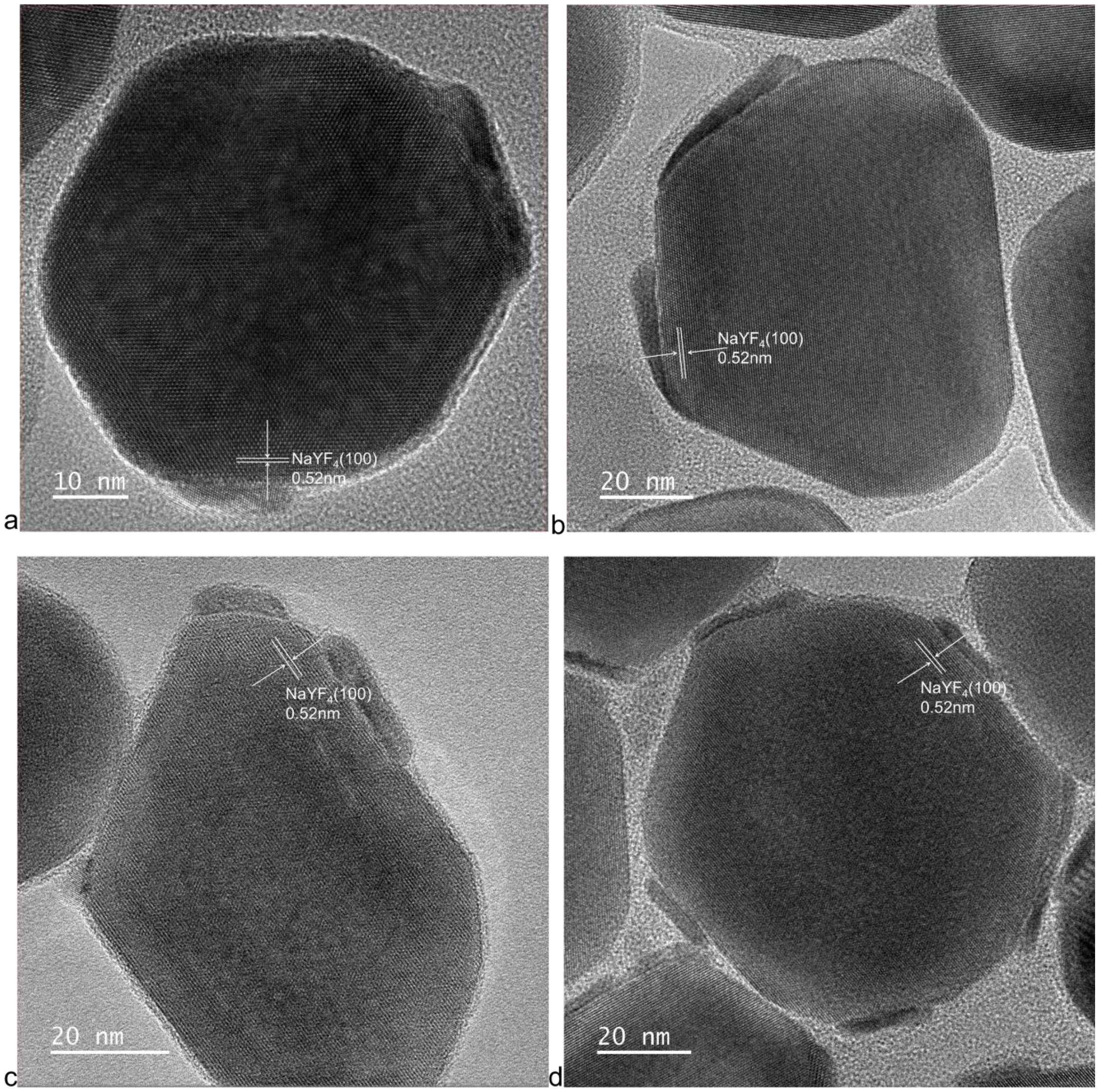

Figure S2. HR-TEM images of $NaYF_4$/YOF HNCs synthesized using different batches consistently demonstrate that YOF selectively grows epitaxially on the {100} facets of $NaYF_4$, confirming the facet-selective growth mechanism.

a

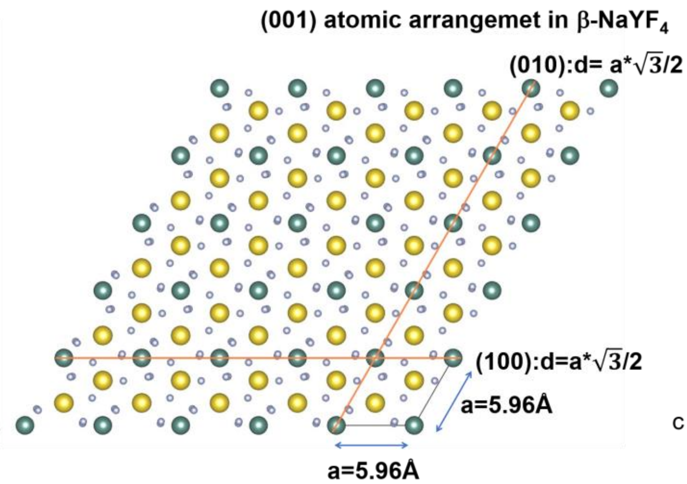

b

	(100)
(010)	60.0º

c

	(100)	(010)
d-spacing	5.2Å	5.2Å

Figure S3. The measurement of the interplane spacings in the HR-TEM in Figure 2c confirms the (100) and (010) facets of $NaYF_4$, so the plane facing the observer is the $NaYF_4$ (001) facet. (a), (b) and (c) are the atomic arrangement of the (100) facets of $NaYF_4$, the angle between specific facets, and the different interplane spacings, respectively, and the HR-TEM measurements are consistent with these data.Obviously, the YOF nanocrystals were grown the {100} family of planes of $NaYF_4$.

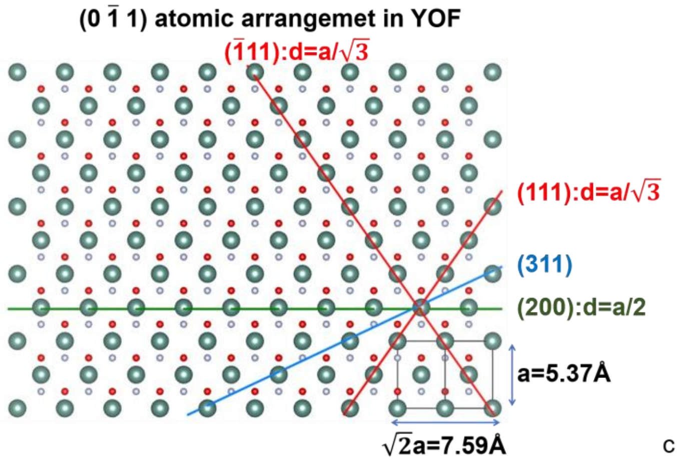

a

b

	(311)
(1̄11)	79.9º
(111)	29.5º
(200)	25.2º

c

	(1̄11)	(111)	(200)
d-spacing	3.1Å	3.1Å	2.7Å

Figure S4. The measurement of the interplane spacings in the HR-TEM in Figure 2a confirms the (200) and (111) facets of YOF, so the plane facing the observer is the YOF ($0\bar{1}1$) facet. (a), (b) and (c) are the atomic arrangement of the ($0\bar{1}1$) facets of YOF, the angle between specific facets, and the different interplane spacings, respectively. From the analysis of the angular relationship of the facets observed by HR-TEM in Figure 2, the (311) facet of YOF is satisfying the condition of being a heterojunction interface.

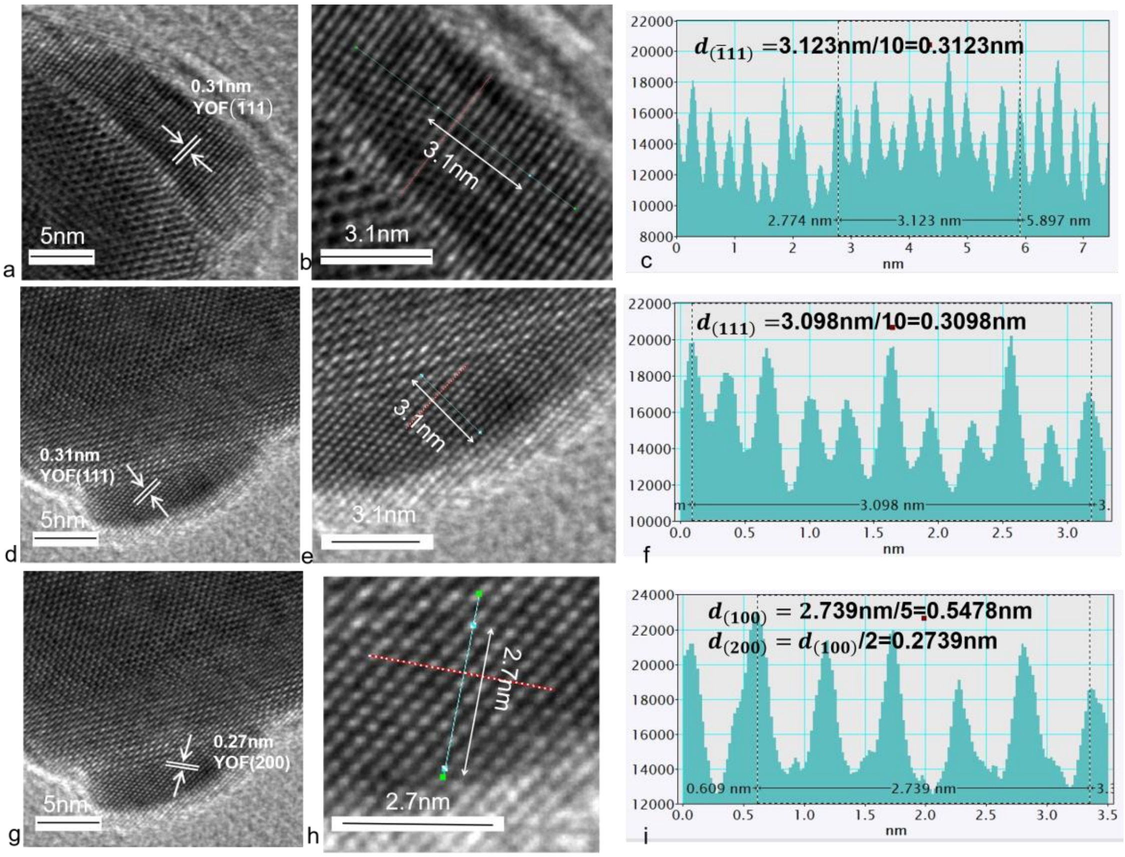

Figure S5. Measurement of the interplane spacings of the YOF in Figure 2 using DigitalMicrograph software. The measured interplane spacings (c, f, and i) are in good agreement with the expected d-spacing of the cubic phase YOF (JCPDS: 06-0346).

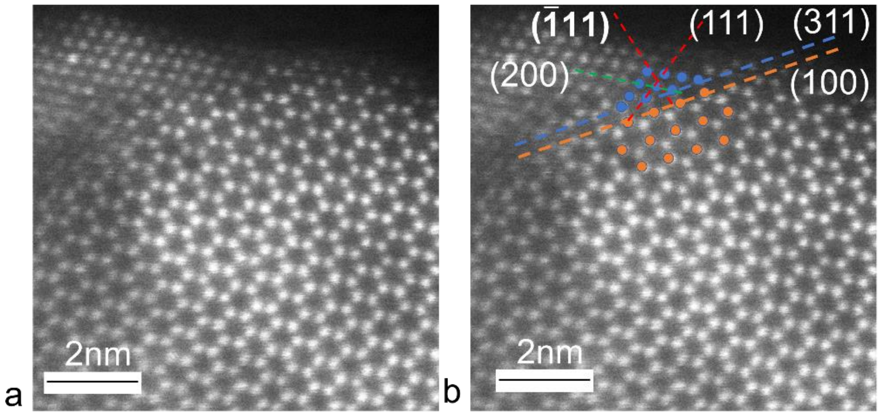

Figure S6. Atomic-resolution HAADF-STEM image (a) was used to determine the interfacial structure (b), which further confirmed that the $NaYF_4$/YOF HNCs heterogeneous interface is composed of a YOF (311) facet and a $NaYF_4$ (100) facet.

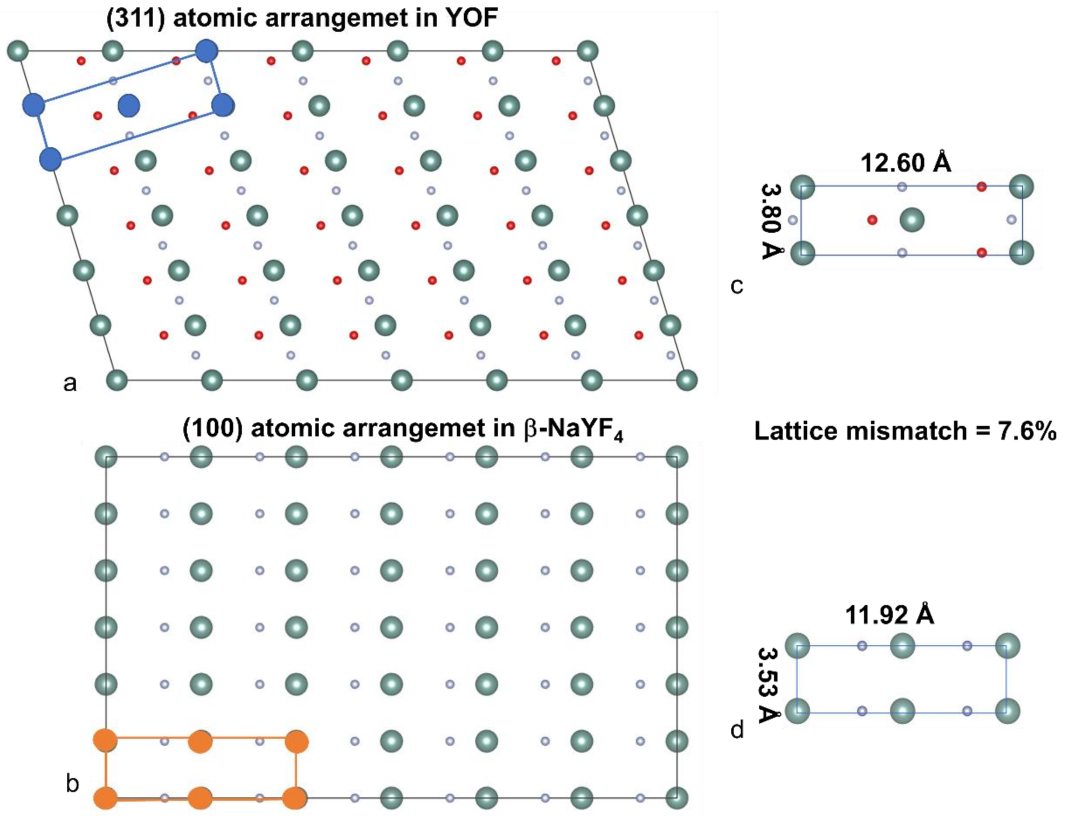

Figure S7. The interface of $NaYF_4$/YOF HNCs consists of the YOF (311) facet with the $NaYF_4$ (100) facet, which was confirmed from the analysis of the HR-TEM image and the atomic-resolution HAADF-STEM image in Figure 2. (a) shows the atomic arrangement of the YOF(311) facets, and (b) shows the atomic arrangement of the $NaYF_4$(100) facets. In order to calculate the lattice mismatch at the interface, we reconstructed the two-dimensional unit cells (c and d) of the two crystal planes with a lattice mismatch of 7.6%.

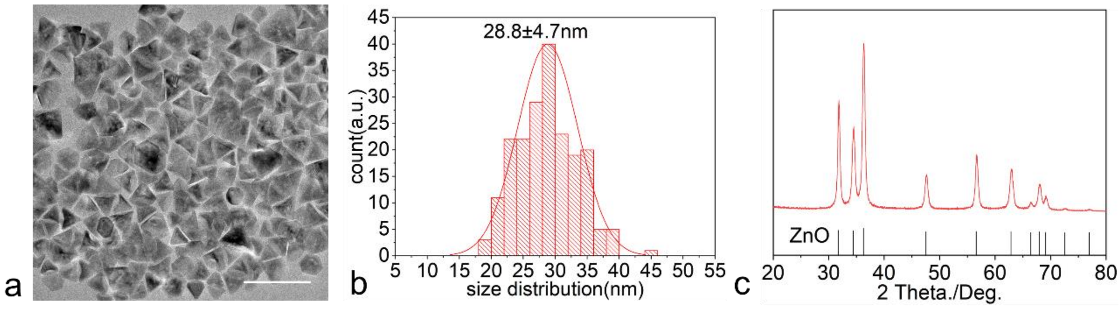

Figure S8. (a) TEM image of the ZnO nanocrystals employed as a sacrificial agent for the synthesis of $NaYF_4$/YOF HNCs, (b) along with the correponding size distribution analysis and (c) X-ray diffraction pattern (JCPDS: 36-1451). Scale bar: 100 nm.

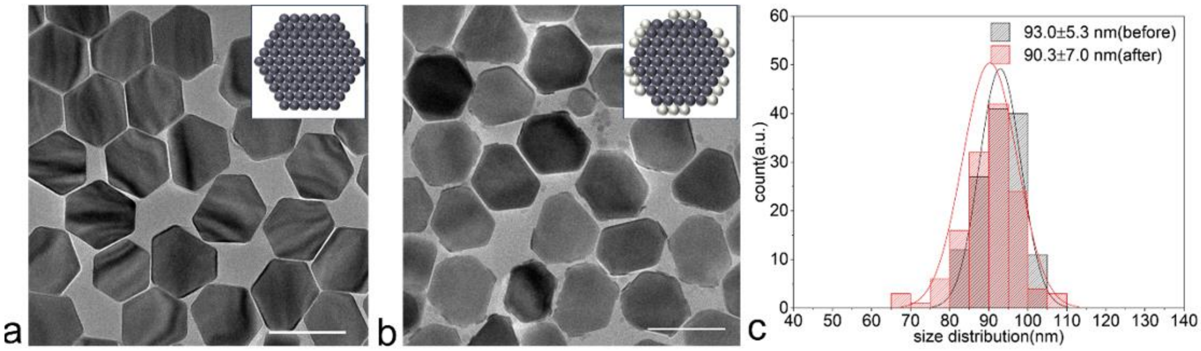

Figure S9. TEM images of $NaYF_4$ nanocrystals before (a) and after (b) YOF growth and the corresponding size distribution (c). The average size of $NaYF_4$ decreases slightly after YOF growth, indicating the presence of dissolution of $NaYF_4$ nanocrystals. Scale bar: 100 nm.

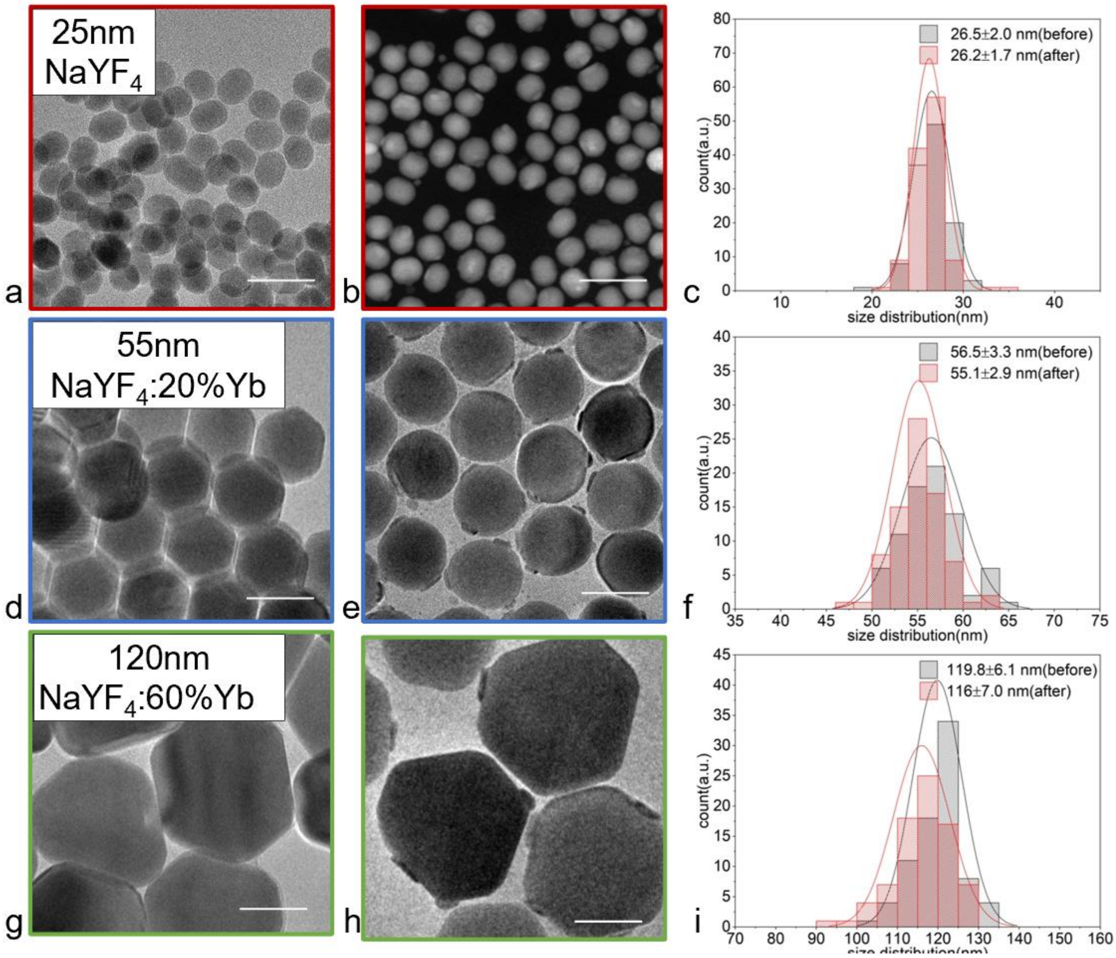

Figure S10. The size of $NaYF_4$ nanocrystals was varied by doping with different ytterbium concentrations. TEM images of $NaYF_4$ nanocrystals of different sizes: 25 nm (a), 55 nm (d) and 120 nm (g), along with their corresponding samples (b: 25 nm, e: 55 nm and h: 120 nm) after the growth of YOF heterostructures. A comparison of the size distribution before and after growth is also shown (c, f and i). $NaYF_4$ nanocrystals of different sizes show a slight decrease in size after YOF growth. Scale bar: 50 nm.

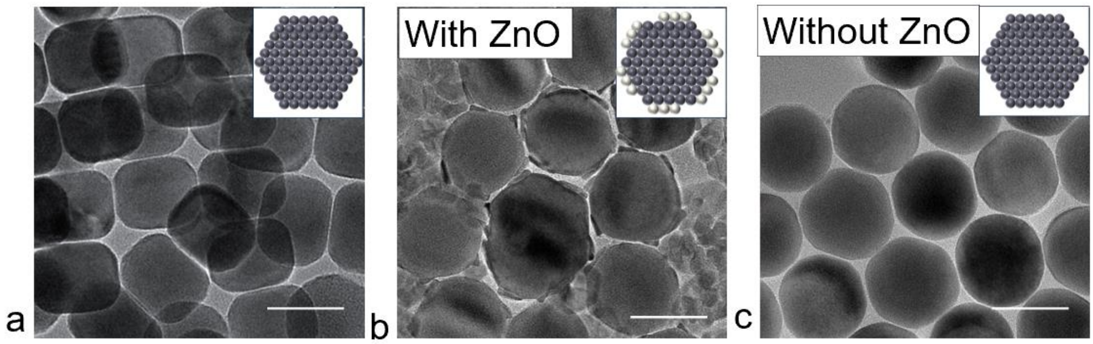

Figrue S11. Illustrating the essential role of ZnO sacrificial agent. TEM images of $NaYF_4$ seeds (a), $NaYF_4$/YOF HNCs with applying ZnO nanocrystals as a sacrificial agent (b) and failure synthesis result of without applying ZnO nanocrystals as a sacrificial agent (c). Scale bars: 50 nm.

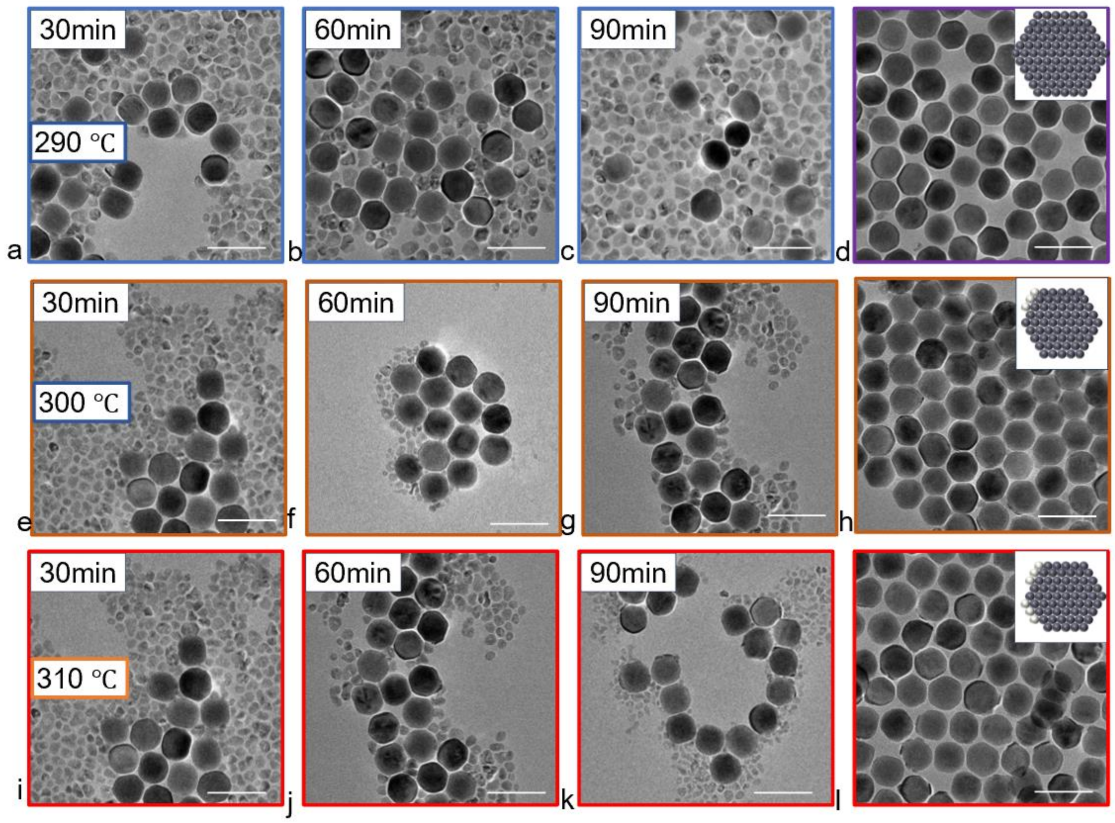

Figure S12. TEM images of $NaYF_4$/YOF HNCs synthesized at 290 °C (a–c), 300 °C (e–g), and 310 °C (i–k) for 30 minutes, 60 minutes and 90 minutes, respectively, as well as TEM images of the final products after ZnO sacrificial agent removal (d, h and l).Scale bars: 100 nm.

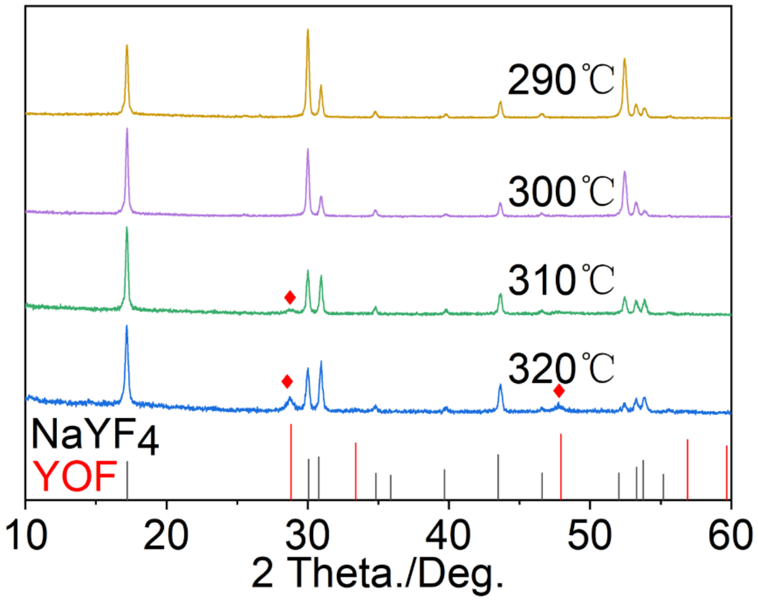

Figure S13. XRD patterns of synthesized $NaYF_4$/YOF HNCs at different temperatures (290 °C, 300 °C, 310 °C and 320 °C). These XRD patterns can be well matched with the reference patterns for the cubic-phase YOF (JCPDS: 06-0346) and the hexagonal-phase $NaYF_4$ (JCPDS: 16-0334).

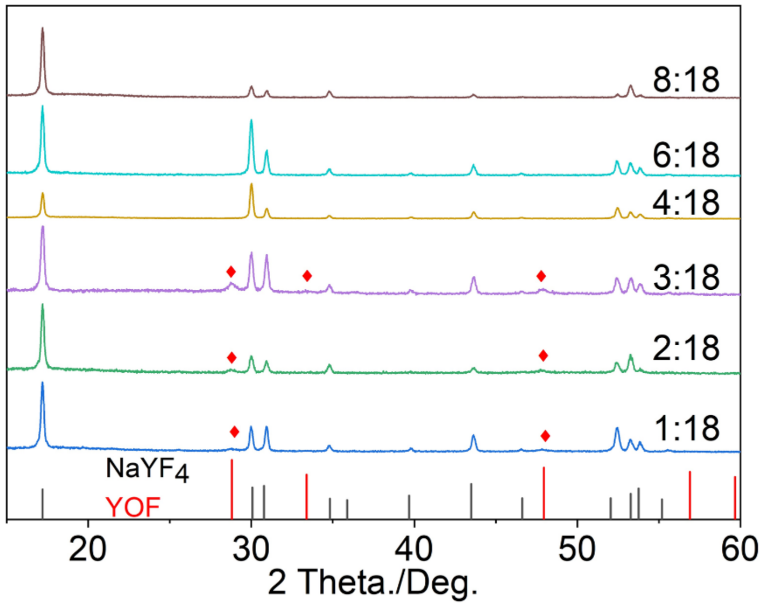

Figures S14. XRD patterns of $NaYF_4$/YOF HNCs synthesized using different oleic acid contents (1 mL, 2 mL, 3 mL, 4 mL, 6 mL, and 8 mL, with a total solvent volume of 18 mL). These XRD patterns can be well matched with the reference patterns for the cubic-phase YOF (JCPDS: 06-0346) and the hexagonal-phase $NaYF_4$ (JCPDS: 16-0334).

(a)

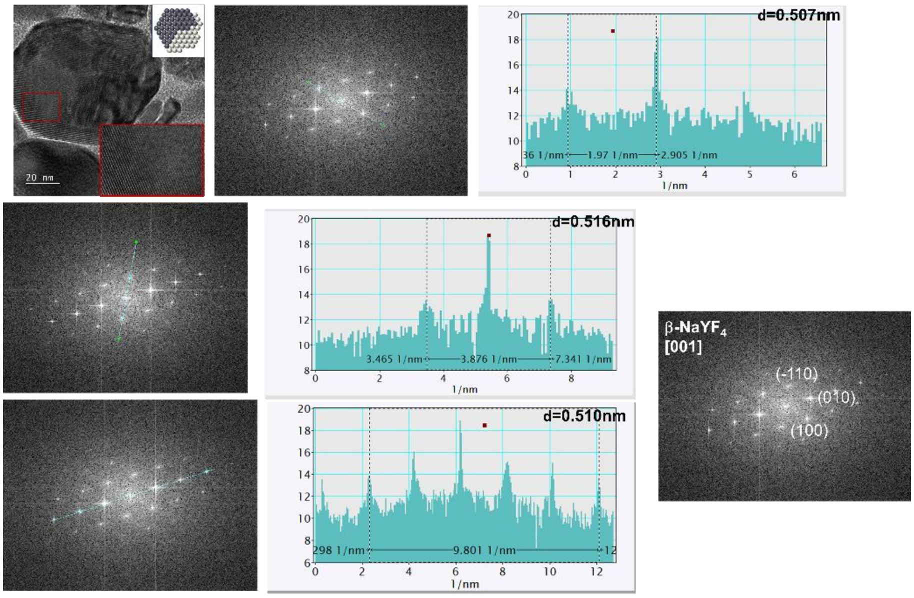

(b)

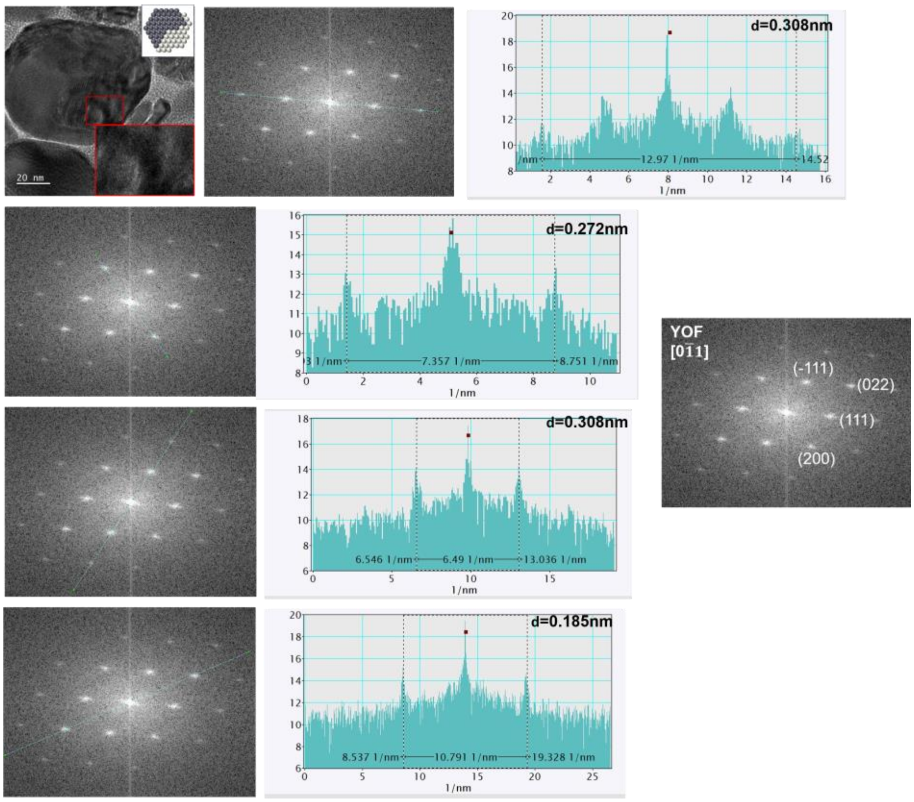

Figure S15. Fast Fourier transform (FFT) analysis was performed on selected regions of the HR-TEM image of single-particle $NaYF_4$/YOF heterostructures (using DigitalMicrograph software). (a) FFT pattern and diffraction spot indexing of the $NaYF_4$ region. (b) FFT pattern and diffraction spot indexing of the YOF region.

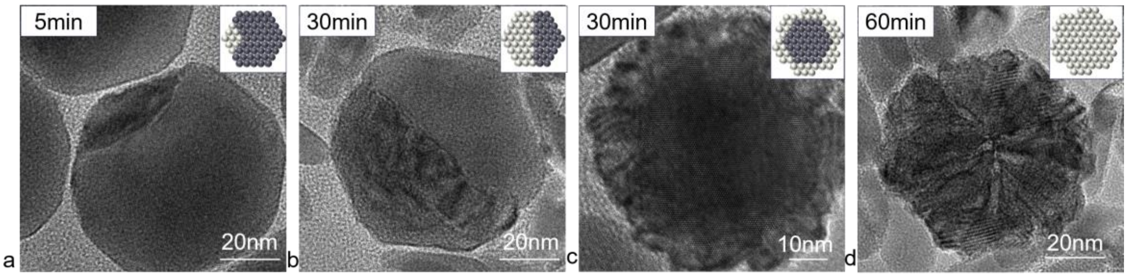

Figure S16. HR-TEM images of $NaYF_4$/YOF HNCs synthesized with Na ions (0.11 mmol/mL) at different reaction times (a: 5 minutes, b and c: 30 minutes, d: 60 minutes).
